# Can Estragole in Fennel Seed Decoctions Really Be Considered a Danger for Human Health? A Fennel Safety Update

**DOI:** 10.1155/2012/860542

**Published:** 2012-07-29

**Authors:** L. Gori, E. Gallo, V. Mascherini, A. Mugelli, A. Vannacci, F. Firenzuoli

**Affiliations:** ^1^Center for Integrative Medicine, Careggi and Department of Preclinical and Clinical Pharmacology, University Hospital of Florence and “M. Aiazzi Mancini”, University of Florence, Viale G. Pieraccini 6, 50139 Florence, Italy; ^2^Interuniversitary Centre of Molecular Medicine and Applied Biophysics (CIMMBA), Department of Pharmacology, University of Florence, 50139 Florence, Italy

## Abstract

Fennel (*Foeniculum vulgare* Mill.) mature fruit (commonly known as seeds) and essential oil of fennel are widely used as flavoring agents in food products such as liqueurs, bread, cheese, and an ingredient of cosmetics and pharmaceutical products. Moreover fennel infusions are the classical decoction for nursing babies to prevent flatulence and colic spasm. Traditionally in Europe and Mediterranean areas fennel is used as antispasmodic, diuretic, anti-inflammatory, analgesic, secretomotor, secretolytic, galactagogue, eye lotion, and antioxidant remedy and integrator. Topically, fennel powder is used as a poultice for snake bites. In Asian cultures fennel was ingested to speed the elimination of poisons. As one of the ancient Saxon people's nine sacred herbs, fennel was credited with the power to cure. Fennel was also valued as a magic herb: in the Middle Ages it was draped over doorways on Midsummer's Eve to protect the household from evil spirits. Recently because of estragole carcinogenicity, fennel has been charged to be dangerous for humans especially if used as decoction for babies. But this allegation do not consider the remedy is prepared as a matrix of substances, and recent researches confirm that pure estragole is inactivated by many substance contained in the decoction.

## 1. Introduction

Fennel (*Foeniculum vulgare* Mill.) belongs to the family of Apiaceae, and is an annual, biennial, or perennial herbaceous plant, depending on the variety, which grows in good soils from sunny mild climatic regions and is a well-known aromatic plant species. *Foeniculum vulgare* has two commercially important fennel types: bitter fennel, *Foeniculum vulgare* Mill. subsp. *vulgare *var.* vulgare*, and sweet fennel *Foeniculum vulgare* subsp. *vulgare *var.* dulce.* Several fennel parts are edible (bulbs, leaves, stalks, and fruits). Mature fruit (commonly known as seeds) and essential oil of fennel are used as flavoring agents in food products such as liqueurs, bread, cheese, and an ingredient of cosmetics and pharmaceutical products. Moreover fennel infusions are the classical decoction for nursing babies to prevent flatulence and colic spasms [1–4]. Traditionally in Europe and Mediterranean areas fennel is used as antispasmodic, diuretic, anti-inflammatory, analgesic, secretomotor, secretolytic, galactagogue, eye lotion, and antioxidant remedy and integrator.

**Figure 1:**
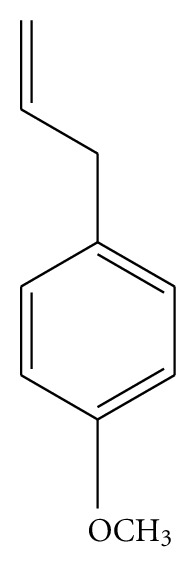
Estragole structure.

It is thus of extreme importance the efficacy, quality, and most of all toxicology of fennel based remedies and preparations is assessed, namely, when estragole (Figure 1), one of its constituents, has been notoriously declared to be a carcinogen substance [5]. 

The European Food Safety Authority (EFSA) suggested the so-called Margin of Exposure (MOE) to be used to set priorities in risk management with respect to compounds that are both genotoxic and carcinogenic [6]. MOE is defined as the ratio between the lower confidence limit of the benchmark dose that gives 10% extra cancer incidence (BMDL10) and the estimated daily intake (EDI) for estragole is estimated from different food sources 0.07 mg/kg bw/day [7]. The MOE for pure estragole amounts to 129–471 and according to EFSA a MOE lower than 10.000 can be considered a priority for human risk [6, 8]. We contend that study of estragole as a single substance can be misleading and misrepresents the activity of this substance when present in the form of a complex herbal extract. This brings into question the validity of studies of pure compoudns that are taken outside of the context of the normal food matrix, which should serve as the benchmark for testing levels in human carcinogenicity studies.

## 2. Chemical Constituents of Fennel

According to the 2nd edition of the European Pharmacopoeia monograph, sweet fennel contains not less than 2.0% v/m of essential oil, calculated with reference to the anhydrous drug. The essential oil is constituted mainly by anethole (80%) (a substance with supposed anticancer properties), it contains not more than 10% estragole and not more than 7.5% fenchone [9]. Other minor constituents may be present including: R-pinene, limonene, *β*-pinene, *β*-myrcene, and p-cymene [9–11]. Furthermore, sweet fennel contains other nonvolatile constituents such as flavonoids and coumarins [12, 13], which have not received till now sufficient attention with regard to pharmacological properties [14].

In a paper the essential oil yield of bitter fennel fruits was 12.5 v/w, whereas 1.8 v/w volatile fraction (corresponding to plant material) was obtained by hydro-distillation of the plant infusion which is equivalent to 14.5% of the initial fennel essential oil. The main constituents of the volatile fraction of the fennel infusion were (hydro-distillation/SPE): trans-anethole (56.4%/58.4%), fenchone (36.2%/39.5%), and estragole (2.5%/2.2%); which were also the major compounds of the genuine bitter fennel essential oil. In infusions the proportion of ethers versus ketones was shifted significantly towards a higher of the latter, compared with the essential oil obtained from the fruits [15].

Generally prepackaged teabags marketed contain unbroken and/or crushed fruit or powdered drug. The use of unbroken fruit to prepare infusions is incorrect: because crushed or powered fruit gradually lose their essential oil content during aging [16], like many herbal remedies.

Many phytochemical researches have been conducted so far to investigate the chemical composition of fennel essential oil with different results: depending on the time of harvests, conservation, region, and area of cultivation. The major components of fennel are phenylpropanoid derivatives: *trans*-anethole and estragole (=  methyl chavicol), and then alpha-phellandrene, limonene, fenchone, and alpha-pinene [17–20].

Essential oil composition depends upon internal and external factors affecting the plant such as genetic structures and ecological conditions; agricultural practices also have critical effects on yield and oil composition in the essential oil crops, although essential oil has some main components that can variate significantly according to the maturation period [21].

Piccaglia and Mariotti [19] indicated the presence of five different chemical groups in the essential oils isolated from fresh aerial parts of wild fennel collected in thirteen Italian areas: (1) trans-anethole, estragole, alpha-phellandrene; (2) *trans*-anethole, alpha-pinene, limonene; (3) estragole, alpha-phellandrene; (4) estragole, alpha-pinene; (5) alpha-phellandrene. About the chemical composition of fennel fruits (=  seeds) the phenylpropanoid fraction (80–89%) and estragole (79–88%), dominated the fruit oil [18]. The relative amount of trans-anethole in these oils were much lower than those that characterize bitter fennel oils [22]. Some previous studies on fennel fruits essential oils have also mentioned estragole chemotypes in variable amounts (a variability in the variety), where estragole alone dominates the oil, or is present together with either trans-anethole or fenchone [18]. These results for the chemical composition of the essential oils of fennel aerial parts and fruits, support the view of Miraldi [7] that knowledge of fennel essential oils is still not enough to distinguish accurately all the existing varieties [18]. So it is very difficult to establish the effective amount of essential oil, estragole, and other substance in different industrial and homemade preparations. In a recent paper [23] was studied the chemical composition of 3 organically cultivated fennel cultivars: *Foeniculum vulgare* var. *azoricum*, var. *dulce* and var. *vulgare. *Gas chromatography/mass spectrometry analysis of the essential oils revealed the presence of 18 major monoterpenoids in all three cultivars but their percentage in each oil were greatly different [23]. The two *azoricum* and *dulce *cultivars are similar in their chemical composition but greatly different than the *vulgare* cultivar: *trans*-anethole accounted for 61% and 46% in the oil of *azoricum* and *dulce* cultivars, respectively, while it accounted for only 5% in the *vulgare* cultivar. Estragole was the major compound in the oil of the *vulgare* cultivar, with a concentration of 58% compared to 12% and 6% in the oils of *azoricum* and *dulce* cultivars, respectively [23]. The essential oils of two of the fennel cultivars, that is, *azoricum* and *dulce*, showed dramatically higher antioxidant activities than the essential oil of the *vulgare* cultivar [23]. The three oils contain similar concentrations of all other major compounds excluding trans-anethole and estragole suggesting that antioxidant activity is mostly related to the concentration of trans-anethole [23]. One of the major differences between the chemical structure of estragole and anethole is the double bond of the propenyl side chain: in anethole is conjugated with the aromatic ring while in estragole it is nonconjugated [23].

## 3. Estragole Carcinogenicity *In Vitro *and Its Metabolic Pathways

For flavonoids formation of reactive intermediates proceeds by their enzymatic and/or chemical oxidation to quinone/quinone methide type metabolites [21], that are reactive alkylating intermediates. For alkenylbenzenes, including estragole, methyleugenol, elemicin, safrole, and myristicin the ultimate carcinogenic metabolites are their 1′-sulfooxy derivatives which degrade to alkylating carbocations that transformed in reactive substance, can give rise to DNA adducts.

Estragole is known to be metabolized along a number of pathways including O-demethylation (to give chavicol), epoxidation of the double bond, 1′-hydroxilation, and oxidative degradation of the side chain to carboxylic acids [24]. Zangouras et al. [24] indicate that at least two pathways, namely, O-demethylation and 1′-hydroxylation exhibit dose-dependency in both mouse and rat. Thus the proportion of the dose that undergoes O-demethylation declines in a dose-dependent fashion and is accompanied by an increase in the proportion of the dose that undergoes urinary elimination [24]. This change presumably arises from saturation of the enzyme systems responsible for O-dealkylation. The corollary of this is that at higher doses a relatively greater substrate level would be available for alternative metabolic reactions such as 1′-hydroxylation [24]. In the mouse the major route of estragole metabolism is via hydroxilation at the 1′ position [20, 25, 26]; producing derivatives with increased carcinogenic potential. Sulfuric acid esters of these compounds have been strongly implicated as the major ultimate electrophilic and carcinogenic metabolites *in vivo*. Thus mouse liver cytosols contain 3′-phosphoadenosine 5′-phosphosulfate-dependent sulfotransferase activity for 1′-hydroxysafrole and 1′-hydroxydehydroestragole [18, 19, 27].

**Figure 2:**
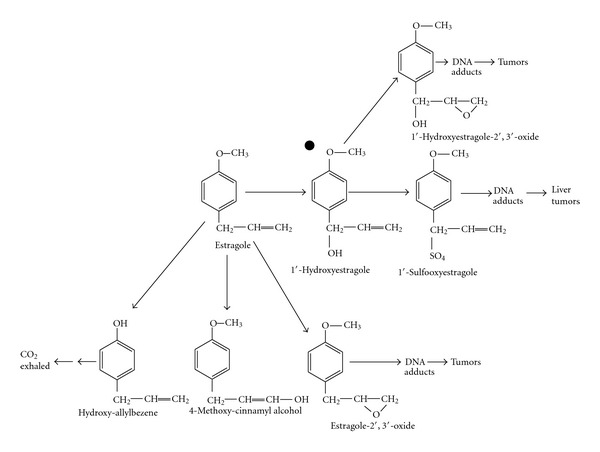
Bioactivation pathway of estragole.

The well-known bioactivation pathway of estragole proceeds by initial metabolic hydroxylation by cytochrome P450 enzymes, leading to the production of the proximate carcinogen 1′-hydroxyestragole, that by involvement of sulfotransferases is converted to the ultimate 1′-sulfooxyestragole; an instable substance that degrades to a reactive carbocation binding to different endogenous nucleophiles and inducing the production of DNA adducts [28], in particular hepatic macromolecular adducts [29]; and these as shown in rodents when given as a pure compound and at high dose-levels-induced hepatomas [30] (Figure 2).

To study bioactivation and detoxification of suspect toxic substance derived from estragole the PBK (Physiologically based kinetic) model was extended to a physiologically based dynamic (PBD) model, by which predict the formation of DNA adducts in the liver of male rats [31]. A PBD model was developed by extending the PBK model through linking the area under the curve for 1′-hydroxyestragole formation predicted by the PBK model to the area under the curve for 1′-hydroxyestragole in *in vitro* incubations with rat hepatocytes exposed to 1′-hydroxyestragole [26]. The PBD model thus obtained, was validated by *in vivo* experimental data on DNA adducts formation in the liver of mice exposed to estragole, since data from rat were not available [26]. Literature reports the formation of 1 adduct in 10.000–15.000 DNA nucleotides after a single i.p. injection of about 400 mg estragole/kg bw/day to female CD-1 mice [32]. At this dose the PBD model predicts the formation of E-3′-N^2^-dGuo, the major estragole DNA adduct formed [33] in the liver of rat at a level amounting to 4 adducts in 10.000 nucleotides. Thus, levels of DNA adducts formation in the two studies are within the same order of magnitude [26]. The slight difference can be explained by the difference in the experimental design of the two studies. At dose levels that match the available estimates for the daily intake of estragole, amounting to 0.01 mg/kg bw [34] and 0.07 mg/kg bw estragole [35], the PBD model predicted amounts of E-3′-N^2^-dGuo DNA adduct formed of, respectively, 2 and 12.8 in 10^8^ nucleotides.

Estragole, like other allylbenzene analogs in the liver, is subject to biotransformation which can generate reactive electrophilic intermediates; the allylic epoxides form readily *in vitro*, but can be rapidly further metabolized to less toxic dihydrol or glutathione conjugates [36]. Epoxide metabolites of allylbenzene are highly reactive and the metabolic pathway initiated by epoxidation has an equivalent potential for biochemical damage to that posed by the 1-hydroxylation pathway [36].

Using levels of epoxides 100-fold the maximal exposure to estragole in human diet in cells of different species, human liver cells had by far the highest allylic epoxide hydrolase activity, seven to 10 times higher than that seen in rat liver; probably the level of physiological protection against these reactants in humans, is higher than in other animal species [36]. Dihydrodiol derivatives were recovered at significant levels in urine of animals fed estragole, so dihydrodiol metabolites presumably represent end products of the epoxidation pathway, and carried out in a test accounted for up to 30% of the total metabolic clearance of estragole [37, 38]; an important outcome because it is approximately the same contribution to the overall metabolic clearance provided by the most studied 1′-hydroxylation pathway.

Recent studies have shown that 1′-hydroxyestragole glucuronide generation is a major pathway of estragole metabolism in rats and mice, which is dose-dependent and accounts for as much as 24% and 33% of the estragole urinary metabolites in rats and mice, respectively [39].

1′-hydroxyestragole and derivated glucuronides are major metabolites formed by human hepatocytes *in vitro*. By 24 h, about 12.5% of estragole is converted to 1′-hydroxyestragole glucuronide by human liver cells [39]. Hence, glucuronidation represents another significant route of detoxification of estragole in all species studied and humans too, that can be activated, although in a different way, by many different flavonoids that are part of the fennel matrix decoction. As shown in the paper of Iyer [39] 1′-hydroxyestragole glucuronidation in 27 individual human liver samples significantly (*P* < 0.05) correlated with the glucuronidation of other UGT2B7 substrates (morphine and ibuprofen). Iyer et al. [39] have determined that 1′-hydroxyestragole, which is the precursor to 1′-sulfooxyestragole, the active metabolite of estragole believed to be carcinogenic, is conjugated mainly by UGT2B7 using cDNA expressed UGT isoforms and correlation studies with other UGT2B7 substrates. UGT1A9 and UGT2B15 were also found to conjugate 1′-hydroxyestragole; this implies that concomitant chronic intake of therapeutic drugs and dietary components that are UGT2B7 and/or UGT1A9 substrates (which are both expressed in the gastrointestinal and liver tissues) may interfere with estragole metabolism [40, 41]. Because the carcinogenicity of 1′-hydroxyestragole is clearly dependent on the balance between formation of the active metabolites, (1′-sulfooxyestragole) and epoxides, and detoxification by glucuronidation; marked interindividual differences in the rate of 1′-hydroxyestragole glucuronidation, may have important toxicogenetic implications. The screen of 1′-hydroxyestragole glucuronidation in liver samples from 27 individuals indicated a significant intersubject variability, with a coefficient of variation of 42% [39].

## 4. The Issue of Estragole Carcinogenicity

Interest in the safety of estragole as a food flavoring stems from observations on the closely related compound safrole, which is both hepatotoxic and hepatocarcinogenic in rodents. Estragole has been shown to be an hepatocarcinogen in preweanling CD-1 mice and preweanling B6C3F1 mice [30, 42]. Administration of 0.23 or 0.46 (w/w) estragole in the diet of CD-1 mice for 12 months resulted in hepatomas in 56 and 71% of the mice [30]. About these results it is probably important to underline that in the first paper [42] the incidence of hepatomas in CD-1 mice (*verum* group), receiving only the vehicle (trioctanoin), was 12%; in a second group [42], 24% of males and 2% of female of CD-1 mice that received trioctanoin were bearing an hepatoma; in another experiment 26% of males that received only trioctanoin by i.p. injection after 12 months had hepatomas, and even 12% of not injected male B6C3F mice developed a hepatoma [30]. We think these data should stimulate reflection about real worth of these experiments in the evaluation of estragole and its derivatives, that probably has been overestimated.

Anthony et al. [27] in his paper reports the metabolism of [^14^C] estragole in rats (by oral intubation) and mice (by i.p. injection) studying the variation of metabolism with dose over the range 50 g to 1000 mg/kg in both species. In mice elimination was essentially complete within 24 hr, and in rats receiving a high dose (500–1000 mg/kg), there was significant excretion on day 2. In both species the main route of elimination of very low doses was exhalation of ^14^CO_2_ and urine was a minor route [27]. In these experiments as the dose level increased, the exhalation of ^14^CO_2_, expressed as a percentage of the dose fell, while excretion in the urine rose. In rats and mice the proportion of urinary ^14^C present as 1′-hydroxyestragole and 4-methoxy-cinnamyl alcohol rose significantly with dose. The excretion of acidic metabolites, indicated by the percentage of urinary ^14^C extracted into ether at pH 1.0 was unaffected by dose size in the mouse and fell in the rat. The elimination of polar unextractable metabolites fell significantly with increasing dose in both species [27]. It is of paramount importance to consider the implications of these results in respect to the papers of Miller and Drinkwater [30, 42], because the dose they administered to animals must be contrasted to the estimated human daily intake of only 70 *μ*g (approximately 1 *μ*g/kg). (Flavor and Extract Manufacturer's Association, 1978).

In fact the hepatocarcinogenicity of estragole in mice has been clearly related to its conversion to 1′-hydroxyestragole, but factors influencing its formation may also cause a related variation in the incidence of tumors and in this context the nonlinear relationship between dose, animal species, and elimination of the 1′-hydroxy metabolite is important [33], particularly in connection with human metabolism. Sangster [25] showed in 2 healthy individuals, administered 1 mg/day of estragole, the excretion of 1′-hydroxyestragole glucuronide in human urine amounts to only 0.3% of the administered dose (0.02 nmol/kg 24 hr), a value far lower than that obtained in rodents even at the lowest doses (0.05 mg/kg body weight; 1′-hydroxyestragole excretion in 24 h in rat 4.5 nmol/kg; in mice 4.5 nmol/kg) [27]. Probably rodent carcinogenicity tests overestimate the risk of estragole carcinogenicity.

Another important difference in estragole metabolism between mice and humans is highlighted by an examination of dose dependency. In this case, the genotoxic metabolite found in urine, 1′-hydroxyestragole, can be used as a indicator of interspecies differences. In mice increasing doses of estragole leads to increasing levels of the metabolite in urine: low doses (0.05–50 mg/kg body weight) led to 1.3–5.4% 1′-hydroxyestragole; high doses (500–1,000 mg/kg body weigh), led to 11.4–13.7% 1′-hydroxyestragole. In humans, the amount of 1′-hydroxyestragole in the urine remained constant at 0.2–0.4% throughout a wide dosage range (1–250 mg estragole or 0.01–5 mg/kg body weight) [25]. A subsequent study on the metabolism of trans-anethole found that it was eliminated by humans 6 to 9 times quicker than by mice [43].

Consideration of these issues (dose, administration form, and differences in metabolism between species) raises doubts about the conclusion that fennel seed can be “reasonably anticipated to be a human carcinogen” [44], It is clear that human and animal metabolism cannot be directly compared but we think data should deserve attention.

In an experiment with male Sprague-Dawley rats (180–200 g) using a CCl_4_ model, using pure fennel essential oil extract was demonstrated a protective effect against the toxicity induced by CCl_4_ in rats. Which constituent(s) of the extract is responsible for this effect was not fully investigated [45]. The anticarcinogenic activity of fennel essential oil considered as a matrix of substance is confirmed by another recent paper using a methanolic fennel extract, that showed a mean ± standard deviation 50% inhibitory concentrations were 50 ± 0.03 *μ*g/mL for the MCF7 breast cancer cell line and 48 ± 022 *μ*g/mL for the Hepg-2 liver cancer cell line. The significant increase in malondialdehyde levels and the significant decrease in catalase activity and glutathione content in liver and tumor tissue in mice bearing Ehrlich ascites carcinoma improved after administration of the extract. *In vitro* pretreatments with fennel essential oil significantly inhibited the frequencies of aberrant metaphases, chromosomal aberrations, micronuclei formation, and cytotoxicity in mouse bone marrow cells induced by cyclophosphamide and also produced a significant reduction of abnormal sperm and antagonized the reduction of cyclophosphamide-induced superoxide dismutase, glutathione, catalase and inhibited increased malondialdehyde activities content in the liver [46]. In a study evaluating the efficacy of a fennel seed methanolic extract for its antioxidant, cytotoxic, and antitumor activities and for its capacity to serve as a nontoxic radioprotector in Swiss albino mice, and on different types of human cell lines *in vitro*, was also assessed the natural antioxidant compounds of the extract for use in industrial application [47]. The extract showed remarkable anticancer potential against a breast cancer cell line (MCF7) and liver cancer cell line (Hepg-2). It also showed strong free radical-scavenging activity (100%). In the conclusions the authors stated that could be used as a safe, effective, and easily accessible source of natural antioxidants to improve the oxidative stability of fatty foods during storage [47].

Nevertheless, has been recently demonstrated a direct carcinogenicity of estragole and found* in vitro* low levels of DNA adducts, with a significant dose response up to 1000 mM, suggesting the possibility of a direct-acting mechanism of adduction [48]. Experiments were also conducted to evaluate the persistence of DNA adducts produced by estragole in V79 cells, after a 25-hour recovery period. The results indicated that adducts are still present after this recovery period, suggesting that at these levels (1000 mM) repair is not efficient. And was shown that estragole did not induce apoptosis in all the assays performed for all concentrations tested, except at the highest concentration of 2000 mM [48]. For this dose and a 24-hour period estragole induced apoptosis to a limited extent, compared with the positive control. The MTT assays also show no significant cytotoxicity (above 50% cellular viability) and the authors concluded that estragole does not induce apoptosis at physiologically relevant doses.

In summary, according to the results obtained, it seems that the genotoxicity of estragole *in vitro* at high doses may ensue in part from direct adduction of DNA which can lead to alkali-labile sites in DNA, resulting in tails in the comet assay, and SCE, due to DNA strand-breaks. Nevertheless, the authors state that doses necessary to induce a genotoxic response are far from physiologically relevant human doses, and therefore the relevance of these adducts for tumor induction in humans *in vivo* needs to be further clarified [48].

## 5. Inhibition of DNA Adduct Formation Inhibition of Carcinogenesis

Recently has been demonstrated that formation of DNA adducts by 1′-hydroxyestragole and cofactor for SULT-mediated conversion could be inhibited by basil extract, the same result was then confirmed in intact human hepatoma cells [49]. This result suggests the likelihood that bioactivation and carcinogenicity may be much lower when estragole is administered at low dose and in a natural matrix.

In experiments using basil derivatives the flavonoid nevadensin, it was able to efficiently inhibit the sulfotransferase-mediated conversion of 1′hydroxy alkenylbenzenes to the corresponding 1′-sulfooxy metabolites responsible for the DNA adduct formation [28]. Further experiments also indicated that nevadensin-mediated inhibition of the formation of the ultimate carcinogenic metabolite of estragole, occurs without reducing the capacity to detoxify 1′-hydroxyestragole via glucuronidation or oxidation [28]. This indicates a potential shift in the phase II metabolism of alkenylbenzenes upon coexposure with nevadensin and/or other flavonoids capable of sulfotransferase inhibition [26]. Assuming a 1% instead of a 100% uptake of nevadensin (similar to a nevadensin: estragole molar ratio of 0.01), the model still predicts about 17% and 43% inhibition of 1′-sulfooxyestragole formation as compared to control in rat and human, respectively [28], so it appears much more active in humans. In the paper of Alhusainy et al. [28] has been shown that at a molar ratio of nevadensin to estragole of 0.06, at which the two compounds are expected to be present in basil, the model predicts an almost complete inhibition of 1′-sulfooxyestragole formation in the liver of male rat and human when assuming 100% uptake of nevadensin.

In the paper of Rietjens [26] even a 1% nevadensin bioavailability at a dose of 50 mg/kg bw of estragole, a dose level in the range of the BMDL10 for tumor formation, dosing of an equimolar quantity of nevadensin, is predicted to result in only 2.4% 1′-sulfooxyestragole formation compared to the amount formed in the uninhibited situation. Our group has isolated and identified nevadensin also in different fennel extracts, so we think nevadensin probably has the same protective effect in fennel extracts too [50].

Moreover using 60 different basil fractions, besides the one identified as nevadensin, about half were able to inhibit SULT activity with different potency [29], and so it can be extrapolated that all together can completely stop SULT activity.

A significant difficulty in evaluating the metabolic, biochemical, and toxicological data for estragole as well as other alkenylbenzenes is that human exposure to these substances results from exposure to a complex mixture of food, spice, and spice oil constituents which may significantly impact the biochemical fate and toxicological risk of the alkenylbenzenes [51].

Recently Alhusainy et al. [51] have shown that given a normal diet may contain a variety of SULT inhibitors, experiments were performed to assess the effect of combined flavonoid exposure on SULT activity as well as on oxidation of 1′-hydroxyestragole to 1′-oxoestragole. To this end a test mixture was defined that mimics a realistic dietary flavonoid mixture and included four flavonoids that were found to be abundant in alkenylbenzene-containing herbs and spices and able to inhibit SULT activity, namely: quercetin, kaempferol, apigenin, and nevadensin, the latter being previously identified as a potent SULT inhibitor present in basil [29]. The compounds were not cytotoxic to HepG2 cells under the conditions used in these experiments and revealed that a significant reduction in the formation of E-3′-N2-dGuo compared to control (no flavonoid(s)) is observed in the human HepG2 cells following coadministration of 50 M of the substrate 1′-hydroxyestragole and 23 M of a flavonoid mixture containing quercetin, kaempferol, myricetin, apigenin, and luteolin (each at a concentration corresponding to its relative contribution in the diet). Altogether, the data indicates a shift metabolism from sulfonation and oxidation to glucuronidation which is a detoxification pathway for 1′-hydroxyestragole [51]. Finally, it is worth noting that even when the concentration of estragole was increased 1000 fold keeping the concentrations of the SULT inhibiting flavonoids at the values defined in the paper, the percentage inhibition of 1′-sulfooxyestragole formation remains the same as obtained at the 1000-fold lower dose of estragole. This is a characteristic of noncompetitive inhibition, where the level of inhibition depends only on the dose of the inhibitors [52].

In our opinion the same effect can be deduced for fennel decoction too, because flavonoids (nevadensin) are a very common substance in plants and can be easily extracted by herb decoction. In fact flavonoids induce detoxifying enzymes such as NAD(P)H: quinone oxidoreductase 1 and glutathione S-transferase which represent important defense mechanism against electrophilic toxicants and oxidative stress [49, 53]. Their prooxidant activity can result in the formation of highly reactive quinone/quinone methide metabolites which fulfill the requirements for electrophilic responsive elements-mediated induction of detoxifying enzymes [26]. It has been demonstrated that the electrophilic responsive elements-mediated response to flavonoids is increased in cells with reduced cellular GSH levels and decreased in cells with increased levels of GSH, supporting a role for the flavonoid quinone/quinone methides in electrophilic responsive elements activation [49, 53]. In infant fennel decoction formulas, the content of estragole was found to range from 241 to 2058 mg L^−1^ in infusions obtained following the same preparation mode (in 100 mL of boiling water) [54]. Authors analyzing these data and taking into account estragole concentration data and applying an approach similar to that used by the ESCO Working Group by a lower estimate of exposure showed the daily consumption of three cups (100 mL) of the tea (2.25 g of comminuted seeds) had the highest estragole level (2058 *μ*g L^−1^, teabag product no. 7; amount of estragole in a tea portion 206 *μ*g) gave place to an exposure of 10 *μ*g/kg bw/day; from this exposure level, they calculated MOE values ranging from 870 to 3210, [54] still a concerning number especially if considered that the decoctions are used for treatment of infant colics. Nevertheless in our opinion because fennel seeds decoctions are a very common remedy used by Italian mothers and if we accept the fact that is an effective hepatocarcinogenic substance, liver pediatric cancer incidence should rise, while in Italy (and in all over the world too) hepatic tumors are extremely rare in children. The Italian official AIRTUM [55] database included only 20 new cases of hepatomas in 1998–2002 in children (age 0–14), corresponding to 1% of incident pediatric neoplasms and incidence trends in 1988–2002 in Italy is −4% [55]. We think these data can confirm that fennel decoction use in infants do not rise significantly the risk of primary liver cancer.

## 6. The Concept of Carcinogenicity

Although international variations in diet and cancer indicate that diet is an important risk factor for many cancers, it has been difficult to ascribe a clear role in cancer causation to exposure to specific individual chemicals or mixture of chemicals [56]. So far, only alcohol intake (cancer of the oral cavity, pharynx, esophagus, and breast) and food contaminated with aflatoxins have clearly been documented as risk factors in humans [57]. Since evidence of carcinogenicity in laboratory animals is generally taken as an indication of potential human carcinogenic hazard, much emphasis is given to the interpretation of findings of animal carcinogenicity and the extrapolation of such findings to humans [56]. The first step in the carcinogenicity hazard identification is to establish whether or not the fennel decoctions are carcinogenic, so we have to establish if we are speaking of pure estragole or a decoction containing estragole and other substances (flavonoids).

Decision about carcinogenicity is generally based on a standard two-year carcinogenicity bioassay in rodents but we think that important evidence should be based on epidemiological data that probably give the definitive answer to the problem. In a recent paper [58] that should be considered a preferred approach to establish carcinogenicity of food basing on data available from animal dose-response analyses and human exposure, has been established by important international bodies (WHO, EFSA, ILSI Europe) a consensus about MOE (margin of exposure) but in the same paper it has been stated that MOE can be used only for prioritisation of risk management actions although the conference stated the difficulty to interpret it in term of real health risk for humans.

There are a number of issues that are central in this step [56]. First, it is important to decide whether the observed tumors in animal experiments are biologically relevant for humans based on the mode of action. So it is fundamental to understand how the toxic substance work, and establish if it is genotoxic or a carcinogen nongenotoxic, the so-called: MOA (mode of action), and site or sites of tumor formation. Second, it must be ascertained whether the existing toxicokinetic and toxicodynamic data are sufficient to reach a definitive conclusion about the likely shape of the dose-response curve for the carcinogenic effect. Especially for food and herbal derivatives it may be particularly difficult. Thirdly, data should be sought, in addition to those from traditional genotoxicity studies, that contribute to an understanding of the mode/mechanism of action. Then any possible influence of nongenotoxic processes, for example, hyperplasia, on the dose-response relationship should be addressed [56]. Finally, it is important to identify data which suggest whether or not there may be one or more subpopulations with special sensitivity/susceptibility to the carcinogenic effect (e.g., dependent on life-stage, gender, and genetic polymorphisms) [56].

Since such judgments in practice almost always rely on animal data, potency estimates are calculated from dose-response information seen in animal experiments, these being surrogates for the human situation [56]. Experimental studies have revealed large variations, of up to 10^8^–10^9^, in the doses of various carcinogenic substances needed to induce tumors in animal experiments [59].

Although hazard identification is a crucial step in the risk characterization process, it is important to recognize that it would be inappropriate to evaluate the toxicity of chemicals solely on the results of hazard identification, based merely on the intrinsic toxicity of the molecule [60]. It happens that data obtained in animals experiments carried out reaching MTD (maximum tolerated dose) may have little biological meaning since they may induce pathophysiological responses that are of little relevance for those that may be the result of much lower doses [60]. A more qualified choice of the dose range in animal studies would lead to a better and meaningful extrapolation process from animals to humans. The key for a correct extrapolation of animal data to humans is the understanding of the mode of action of chemicals. Unfortunately, this is not always the case, like is the case of d-limonene and formaldehyde. D-Limonene is recognized as an experimental carcinogen because causes nephropathy and kidney tumors in male rats, through binding to *α*_2u_-globulin in the kidney; but it is a globulin male rat specific and do not represent any risk for human health [60, 61]. Formaldheyde has been classified as a known human carcinogen, causing several cancer, and particularly nasopharyngeal cancer and leukemia, but innocuous if added to milk as a bacteriostatic, because is rapidly transformed in spinacine, an innocuous substance [62].

Traditionally, an uncertainly factor of 100 is used, based on a 10-fold factor to allow for differences between average humans and a 10-fold factor to allow for differences between average humans and sensitive individuals [60]. A “false negative” decision about the carcinogenicity of a substance occurs when the bioassay fails to produce a statistically significant increased tumor incidence when in fact the chemical truly causes an increase in the tumor incidence at the dose tested. This is a statistical limitation resulting from the number of animals (generally 50) used per species-sex-dose group. Using the estimate of the dose-response trend obtained from other studies for each specified tumor type/tissue site in animals and the standard error of the trend, it is possible to estimate the approximate probability (power) of detecting a statistically significant trend only as a function of the sample size [63]. But if much more animals are used per dose group the statistical analysis could change the results and a substance can be categorized as carcinogenic, only because the sample size is changed [63].

## 7. Conclusion

In all of the animal studies reviewed, isolated, purified estragole was used. Thus the findings give a toxicological profile of this only molecule and not the profile risk of the entire decoction. In humans estragole usually enters the body as a component of fennel tea, or as a food that has been seasoned with herb that contains many other substance like nevadensin, epigallocatechine, other flavonoids, and anethole, that have a protective role and so counterbalance to the possible effect of pure estragole. In this context estragole occurs in the form of an extremely complex phytochemical mixture. If data about single constituent *in vivo* can be used as basis for statements about a herb, then data about other constituents should also be fully considered, because we think it is the only way to establish definitively if a substance is dangerous or not; and if it is a substance used from many years and in particular subsets of consumers or patients epidemiological data, when available, can help in establishing, together with the real mode of use, the effective risk for consumers.
